# Very large hidden genetic diversity in one single tumor: evidence for tumors-in-tumor

**DOI:** 10.1093/nsr/nwac250

**Published:** 2022-11-11

**Authors:** Bingjie Chen, Xianrui Wu, Yongsen Ruan, Yulin Zhang, Qichun Cai, Luis Zapata, Chung-I Wu, Ping Lan, Haijun Wen

**Affiliations:** State Key Laboratory of Biocontrol, School of Life Sciences, Sun Yat-sen University, Guangzhou510275, China; GMU-GIBH Joint School of Life Sciences, Guangzhou Medical University, Guangzhou 511436, China; Evolutionary Genomics and Modelling Lab, Centre for Evolution and Cancer, The Institute of Cancer Research, London SW7 3RP, UK; Department of Colorectal Surgery, The Sixth Affiliated Hospital, Sun Yat-sen University, Guangzhou 510655, China; Guangdong Provincial Key Laboratory of Colorectal and Pelvic Floor Diseases, The Sixth Affiliated Hospital, Sun Yat-sen University, Guangzhou 510655, China; State Key Laboratory of Biocontrol, School of Life Sciences, Sun Yat-sen University, Guangzhou510275, China; State Key Laboratory of Biocontrol, School of Life Sciences, Sun Yat-sen University, Guangzhou510275, China; Cancer Center, Clifford Hospital, Jinan University, Guangzhou 511495, China; Evolutionary Genomics and Modelling Lab, Centre for Evolution and Cancer, The Institute of Cancer Research, London SW7 3RP, UK; State Key Laboratory of Biocontrol, School of Life Sciences, Sun Yat-sen University, Guangzhou510275, China; Department of Colorectal Surgery, The Sixth Affiliated Hospital, Sun Yat-sen University, Guangzhou 510655, China; Guangdong Provincial Key Laboratory of Colorectal and Pelvic Floor Diseases, The Sixth Affiliated Hospital, Sun Yat-sen University, Guangzhou 510655, China; State Key Laboratory of Biocontrol, School of Life Sciences, Sun Yat-sen University, Guangzhou510275, China

**Keywords:** intratumoral heterogeneity, tumor origins, cancer evolution, tumors-in-tumor

## Abstract

Despite the concern of within-tumor genetic diversity, this diversity is in fact limited by the kinship among cells in the tumor. Indeed, genomic studies have amply supported the ‘Nowell dogma’ whereby cells of the same tumor descend from a single progenitor cell. In parallel, genomic data also suggest that the diversity could be >10-fold larger if tumor cells are of multiple origins. We develop an evolutionary hypothesis that a single tumor may often harbor multiple cell clones of independent origins, but only one would be large enough to be detected. To test the hypothesis, we search for independent tumors within a larger one (or tumors-in-tumor). Very high density sampling was done on two cases of colon tumors. Case 1 indeed has 13 independent clones of disparate sizes, many having heavy mutation burdens and potentially highly tumorigenic. In Case 2, despite a very intensive search, only two small independent clones could be found. The two cases show very similar movements and metastasis of the dominant clone. Cells initially move actively in the expanding tumor but become nearly immobile in late stages. In conclusion, tumors-in-tumor are plausible but could be very demanding to find. Despite their small sizes, they can enhance the within-tumor diversity by orders of magnitude. Such increases may contribute to the missing genetic diversity associated with the resistance to cancer therapy.

## INTRODUCTION

Drug resistance in tumors has been attributed to the genetic diversity among cells within a single tumor [1–3]. Sequencing of multi-regional samples has indeed uncovered extensive genomic diversity [4–9]. However, the genetic diversity within each tumor is still limited due to the close genealogical relationship between cells.

The view that each tumor mass originates from one progenitor cell is cogently presented in Nowell's seminal paper—a view to be referred to as the Nowell dogma [10]. Cancer genomic data have amply corroborated the Nowell dogma by showing thousands of mutations shared by cells of the same tumor. Given the limited genealogical depth, the maximal divergence between any two clones is modest. For example, while Ling *et al.* [4] show hundreds of millions of nucleotide variants (clonal and subclonal) within a single tumor, no pair of clones differ by >10 non-synonymous changes and a pair of randomly chosen cells usually differ by <5. Most importantly, these cell clones generally do not deviate from the expectation of fitness neutrality. In other words, variants derived from a common ancestor mostly behave like neutral noises with little functional significance [4–9].

In contrast to the genomic analyses, which do not find much adaptive diversity, empirical studies often encounter drug resistance and, in essence, adaptive diversity [1–3]. Such apparent contradiction hints to the existence of ‘missing diversity’. An often-discussed possibility is that the tumor microenvironment (TME) creates spatial heterogeneity and promotes clonal diversity [11–13]. This conjecture assumes sufficient heterogeneity in TME as well as adaptive diversity among cells, neither of which would be easy to prove.

The issue of limited diversity due to the single origin can be resolved if the tumor in fact harbors multiple cell clones of independent origins but all, except the dominant clone, are too small to detect. In particular, the emergence of tumors may indicate certain qualities of the local TME conducive for multiple origins. With clones of multiple independent origins, the maximal diversity between cells can easily be >200 coding mutations, as can be inferred from tumors among individuals [14,15]. This level of divergence is far greater than that between two cell clones of the same tumor, usually with <10 coding mutations.

We wish to test whether the missing genetic diversity can be explained by multiple clonal origins within the same tumor, which shall be referred to as ‘tumors-in-tumor’. In the following section, we present the theoretical conditions (e.g. cell fitness, spatial patterns of clones, etc.) under which tumors-in-tumor may be realized. Nevertheless, even when the conditions are met, we find the detection would still require nearly-exhaustive sampling. In the next section, we test the theoretical conjecture by dissecting two cases of colorectal cancer. Both the theory and the observations may potentially be of high clinical significance.

## RESULTS

### The theory of tumors-in-tumor

At about the same time as Nowell's proposal, Cairns [16] in another seminal study suggested that each multicellular organism is compartmentalized into numerous small units, usually in the form of stem-cell niches. With the compartmentalization, multiple occurrences of clonal expansions in the same tissue would be plausible. Recent studies have shown that small patches of normal tissues often harbor a large number of cellular clones of various sizes [17–21]. A sizable literature also exists on tumors of independent origins abutting each other [22–27]. The interplay between TME and mutant cells would be the basis of the concept of field cancerization [28], which prescribes a tissue architecture that facilitates multiple originations of neoplasia and tumors.

In this tissue architecture, the evolutionary unit of clonal expansion and tumorigenesis is the stem-cell niche. In the colon, this niche would be the crypt [29–31]. Given tens of millions of crypts in the human gut, each local patch with a suitable TME may have thousands of neoplasia and proto-tumors. The theoretical question is the fates of these many cell clones. Since they are not likely to be identical in fitness by having different sets of mutations, they would compete by the ‘winner-takes-all’ rule. In this conventional view, there would be only one winning clone in the end.

**Figure 1.**
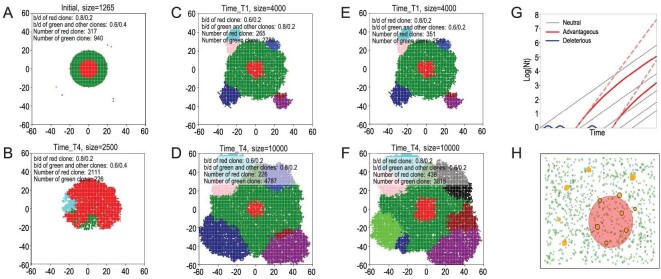
The growth dynamics of competing tumors and the possibility of ‘tumors-in-tumor’. (A) The initial state of Rows 2 and 3. Different colors designate independently-emerged cell clones (ICCs) of a small area. The green clone is surrounded by a main clone colored in red with other clones scattered nearby. The red clone differs from the green and other clones in birth and death rate. (B) If the inside red clone has a selective advantage above a threshold, it would break out by engulfing and then eliminating the weaker green clone outside. (C and D) The clonal states at different time points when the total cell number is 4000 or 10 000. The red clone inside (birth rate at 0.6) is less fit than the green clone on the outside (birth rate at 0.8) with the death rate at 0.2 for both. (E and F) These two panels, the mirror images of (C) and (D), show the stronger red clone inside. It is most interesting to contrast the two rows of (C) and (D) vs. (E) and (F) that have remarkably similar dynamics despite the reversal in the selective advantage. This is because the selective coefficient (either positive or negative) is diluted to *N*^(1/3)^ of the initial value. (G) Tumor mass in log scale ($ log( {Nt}) $ is linear if the clones grow exponentially (black lines). Red lines indicate advantageous clones that are much rarer than the neutral ones initially. Dotted red lines indicate exponential growth in a spatially panmictic population as in leukemia. In solid tumors, after the initial rapid growth, the fitness advantage gradually diminishes and approaches the rate of the neutral clones (solid red lines; see the text). The blue lines indicate clones less fit than the neutral ones, which would eventually disappear. (H) This panel illustrates another aspect of clonal competition when tumors are abutting each other without one being enclosed by the other. Hence, the clones grow more or less independently and the lesser ones are not eliminated. This coexistence may explain the dynamics of multifocal tumors in the literature.

We first illustrate the outcome of cell competition in Fig. 1A and B, based on a spatial Moran model of population genetics. The simulations start with clones in different colors shown in Fig. 1A where a main clone colored in red is nested inside another clone colored in green. In Fig. 1B, the fitness advantage of the inside clone (red) is large enough to break through the barrier of the outside clone to proliferate. Figure 1B thus corroborates the conventional view of ‘the stronger always win’. In this view, most tumors should harbor only one winning clone at the end [4,6,32,33].

The ‘winner-takes-all’ view is valid in the conventional Wright–Fisher model of spatial panmixia whereby different genotypes are thoroughly mixed in the population. As a result, the fitness differences are fully realized. In a more realistic setting portrayed by the spatial Moran model, neighboring cells in a solid tumor are usually of the same genotype as they have just descended from a recent common ancestor. We shall call these cells ‘selves’ as opposed to non-selves from a different ancestry in a different part of the tumor.

Imagine a clone of selves to be spherical with a diameter size of *L* cells. For this clone, the proportion of cells exposed to the competition with non-selves would be only on the periphery, accounting for $\frac{1}{L} = \frac{{{L}^2}}{{{L}^3}}$ of the total cell mass. Therefore, when *L* is very small, nearly all cells are under selection. However, even when *L* is only 10 and the clone has 1000 cells, the fitness advantage (or disadvantage) would be only 10% (=1/*L*) of what it should have been. With localized competition, the fitness of the clones would be *s*/*N*^1/3^, where *s* is the selective advantage and *N* is the number of cells of the clone. As a result, an advantageous mutation would approach fitness neutrality when the tumor grows to merely a modest size.

In Fig. 1, we use a range of parameter values to show the two possible outcomes of the competition. In Fig. 1B, the enclosed clone is able to break through the siege as its selective advantage is sufficiently large. In Fig. 1C–F, the enclosed clone is bottled inside, regardless of whether it is more or less fit than the clone surrounding it. In Fig. 1C and D, the red clone inside (birth rate at 0.6) is less fit than the green clone on the outside (birth rate at 0.8) with the death rate at 0.2 for both. In Fig. 1E and F, the birth rates are reversed with the growth advantage going to the red clone inside. However, the patterns are nearly identical. Clones on the outside grow continually and the red clone enclosed inside grows slowly although the red clone does become slightly bigger in the third than in the second row. Indeed, when a clone is nested inside another one, the selective advantage (or disadvantage) becomes vanishingly small as tumors grow. This is the general pattern found in a large parameter space.

Figure 1G illustrates the growth dynamics of advantageous clones. The simple figure explains the various patterns seen in Fig. 1A–F. Under full competition, the selective advantage, *s*, is constant and the slope of the dotted red line would dictate the fate of each clone. With localized competition, the fitness of the clones would be *s*/*N*^1/3^, as stated above. The slope of the red solid line would eventually approach that of the neutral clone (black line). Whether a bottled-in but advantageous clone could break through the siege would depend on a number of parameters. Some of the parameters such as their relative clonal size and spatial relationships are ecological in nature while others (e.g. their fitness differential) are genetic. The opposite trend may often be true too. For similar reasons, a weaker clone inside may persist for a long time, since the effective selective force diminishes as the tumors grow.

In conclusion, multiple tumors may sometimes (or often, but not always) coexist as tumors-in-tumor even when these clones are unequal in fitness. A corollary of the theory is that the engulfed clones would tend to be very small and difficult to detect, as illustrated in Fig. 1H (see legends). The bottled-in clones may not be threatening to the patient, unless the surrounding clone is destroyed by, say, therapeutic means.

### Experimental test of the hypothesis of tumors-in-tumor (coexistence of multiple-origin clones)

The objective in this section is to search for tumors-in-tumor that are expected to augment the genealogical depth (and, hence, the genetic heterogeneity) of the tumor by >10-fold as stated in the ‘Introduction’.

Operationally, independent clones are defined as those with non-overlapping sets of somatic mutations. A tumor (within another tumor) is defined by a cell mass meeting these criteria: (i) burdened by a larger mutation load than normal tissues [18–21,34]; (ii) being a much larger mass than those reported for normal cell clones; and (iii) able to disperse non-locally within the larger tumor. Clones not meeting these criteria are considered non-cancerous or pre-cancerous. The issue will be revisited in the ‘Discussion’ section.

To find tumors-in-tumor, the conventional practice of analysing a large number of tumors with a few (as small as one) samples per tumor would not be suitable. Instead, it is necessary to analyse a large number of samples from each tumor and the sample volume has to be small. A further element would be the choice of tumors at the right evolutionary stage. In the main case (Case 1), the primary colon tumor yields 145 microdissected samples (see ‘Methods’ section and Supplementary Fig. S1), 7 of which are subjected to whole-genome sequencing (WGS). We chose 106 variants for validation among the remaining 138 samples (see ‘Methods’ section). The same dense sampling strategy is also applied to the four liver metastatic lesions (unpublished results). In Case 2, 9 WGS samples and 401 target sequencing samples (including 209 from primary tumor) were taken from the primary and metastatic tumors (colon and liver, respectively).

#### Observations on Case 1: multiple independent clones in the same tumor

**Figure 2.**
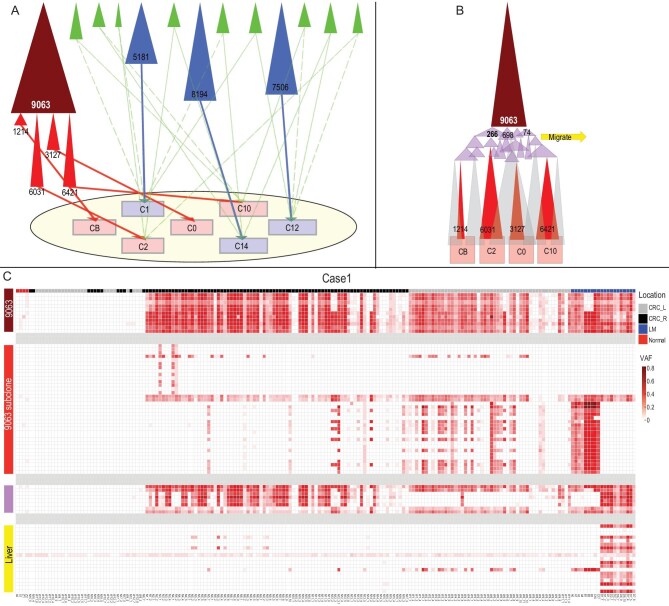
The clonal composition of the Case 1 tumor revealed by WGS and target validation. (A) Clones identified in Table 1 are represented by shaded triangles, named by the number of mutations associated with the clone. The clones are shaded in five different colors: maroon, red, pink (which form a family), blue and green. The distribution of these clones among the seven WGS samples from the tumor (the oval) are shown by the thin lines. The four red clones are all subclones of the parent Clone 9063. Note the presence of 13 independent clones consisting of a dominant clone (represented by maroon and its red subclones), 3 large blue clones and 9 small green clones (see Table 1). Cells of the small green clones are dispersed, presumably by the dominant maroon clone as it expands. (B) The widely distributed maroon clone spawns the four sample-specific red clones via a large number of pink clones, which represent a stage of geographical expansion of the maroon clone. Hence, the pink clones are less localized than the larger red subclones. The approximate timing of the metastasis to the liver is indicated by the block yellow arrow at the pink-clone stage. (C) Mapping the distribution of the maroon, red and pink clones across the 138 samples from the primary tumor and the liver metastases. Each column represents a sample. labeled by different colors (black, orange and cyan) on the top row of the heat map to show their locations. Each row represents the distribution of an SNV across the 138 samples. The collection of 106 SNVs are separated into four groups (maroon, red and pink as shown in Fig. 2A plus the yellow group, representing mutations of the liver metastasis). The color intensity in each box indicates the frequency of the SNV in each sample.

*WGS data from seven major samples reveal 13 independent clones in the primary tumor.* The starting point of Case 1 is the WGS sequencing data from seven samples (labeled C0, C1, C2, etc.) of the primary tumor. The data permit the identification of 13 independently originated clones. These 13 clones fall into three categories that are color-coded in ‘reddish’, blue and green, respectively, in Table 1. Each clone will be identified by the number of mutations carried by that clone alone. We summarize the clonal patterns in Fig. 2 with the locations of the samples (Fig. 2A). Given the complexity, the deduction of the clonal relationships of Fig. 2 from the data of Table 1 is given in details in Supplementary data. Below, we explain the patterns of Fig. 2.

**Table 1.** Grouping of mutations into clones in Case 1.

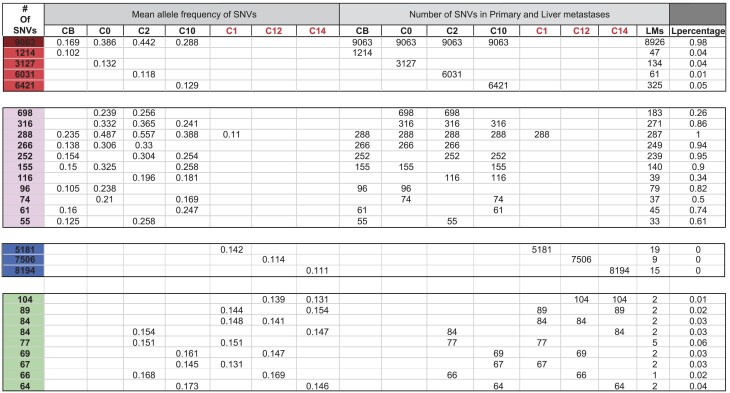	

The single-nucleotide variations (SNVs) in the 7 whole-genome sequencing (WGS) samples of Case1 are grouped according to the clonal patterns. Each clone, representing a group of mutations, is named by the number of mutations associated with the clone. Each row represents such a clone. Clones having similar geographical patterns are further partitioned colored categories, reddish (maroon, red and pink), blue and green. Columns of the table are divided into three sections: (1) Clonal frequency in the seven WGS samples; (2) number of mutations in these seven samples; (3) number of mutations in the samples from the liver metastases; and (4) (the last two columns) the number and proportion of the mutations observed in the liver metastases (L denoting the metastases).

Category I (‘reddish’ clones that have three shades)—In this category, there is only one major clone (Clone 9063; dark red) that then splits into four major subclones (Subclones 1214, 3127, 6031, 6421; bright red). As shown in Fig. 2A, each subclone is mainly associated with a single sample. For example, the Subclone 1214 is found in the CB sample. Figure 2B shows how these four subclones are derived from the parent Clone 9063 that spread widely over the entire tumor (see cell movement in the next section). As this parent clone moves, it accumulates additional mutations (the pink group of mutations of Table 1) that are inherited by the red clones later. Note that the pink group of mutations emerged when the parent Clone 9063 was still moving about in the tumor. Also, as indicated by the yellow arrow of Fig. 2B, it is during the emergence of the pink group of mutations when the metastasis to the liver took place.

Category II (the three blue clones)—The major Clone 9063 likely exhibits the characteristics of the dominant clone of most tumors. Hence, the most intriguing clones found in this study are the three blue-coded clones of Fig. 2A. The numbers of mutations accumulated are rather high (5000–8000) and the clone size is not trivial. A possible explanation is that they correspond to the bottled-in clones (see the interior clones of Fig. 1). These three clones may possibly be highly tumorigenic but were besieged by the dominant Clone 9063, thus failing to break out.

Category III (the nine green clones)—These clones appear to be somewhere between clonal expansions in normal tissues [18–20] and true tumors. These clonal expansions are detected when the cells are found in different samples. For example, Clone 84 is found in C12 and C1 while Clone 66 is present in C12 and C2 (see the thin green arrows in Fig. 2A).

In short, this primary tumor is composed of at least 13 clones—one large, three medium-sized and at least nine small clones.

**Figure 3.**
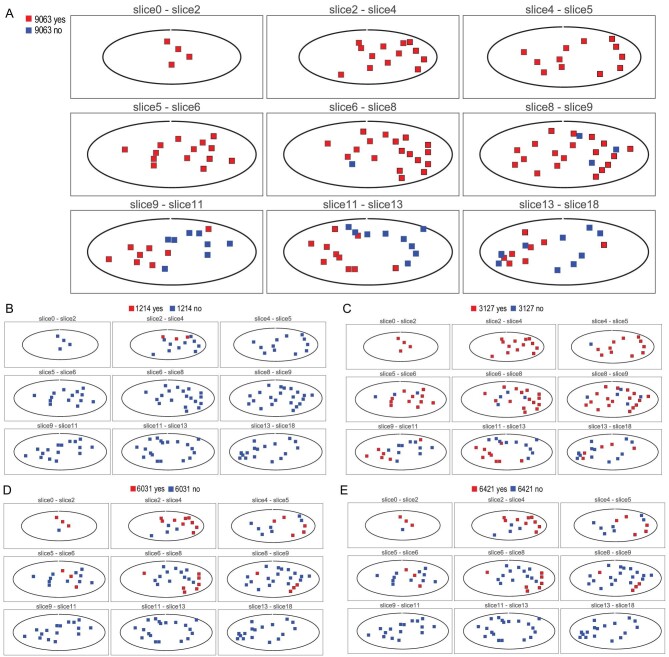
The 3D distributions of SNVs across 138 samples from nine serial planes in Case 1. Each of the five groups of mutations is shown as present (red) or absent (blue) among the nine planes (one plane for each panel) of the tumor. Each plane represents a composite of two or three neighboring slices from the 18 serial slices of the tumor as illustrated in Supplementary Fig. S1. (A) Note that the SNVs from the major Clone 9063 (maroon in Fig. 2) are not found in many samples of the lower three planes. The geographical distribution of SNVs from the four red clones (the subclones of the parental Clone 9063) are showed from (B to E). These subclones are indeed strongly localized, supporting the interpretation given in Fig. 2.

*Wide-ranging cell movement becomes locally constrained as tumor evolves.* We now inspect the movements of cells carrying the dominant clone mutations (i.e. those marked by dark red or bright red color). As these cells spread, they should also be locally competitive if they succeed in containing other emerging clones (like the blue clones). Here, we genotype 138 samples from the primary tumor using 106 single-nucleotide variations (SNVs) of Clone 9063 (dark red) as well as the four subclones (bright red) as shown in Fig. 2C to track cell movement. In Fig. 3, the tumor is portrayed in nine panels, each of which is a composite of two or three neighboring slices from the 18 slices of the tumor. The middle slice of the fifth panel in Fig. 3A is the pattern of Fig. 2. The 3D distribution of Clone 9063 and the four subclones is portrayed by a group of nine panels.

In Table 1, Clone 9063 is shown to populate 80% of the samples all over the tumor. The 3D distribution of Clone 9063 can be visualized in Fig. 3A with red squares indicating the presence and blue for absence. This clone occupies the top portion of the tumor completely but has not reached the lower portion and it shows contiguity in its spread, as can be reconstructed from the 18 slices. Figure 2A, being a slice in the middle (the slice for WGS), happens to present the average prevalence of Clone 9063. In short, the dominant clone does not account for the entire tumor mass. The four subclones (red clones in Fig. 2 and Table 1) are all specific to each of the four samples shown in Fig. 2. Within the entire tumor (see Fig. 3B–E, each having nine panels), they exhibit a range of characteristics. The Subclone 3127 of Fig. 3C is the most broadly distributed but it still occurs in <50% of the samples (note that the presence of the clone is shown with a red dot even at a very low frequency). The remaining three subclones are all rather locally distributed, this being particularly evident for Clone 1214.

We interpret the mutations of these subclones to have emerged rather late during the tumor growth. At the late stages, cells do not get pushed around by clonal expansion and thus result in the much more highly localized distributions. In conclusion, the parent clone ranges very widely but, as it evolves by accumulating more mutations, the subclones become more constrained spatially. The number of mutations each clone accrues should also be a measure of the passage of time. In short, most of the cells of these clones are constrained in a localized area for a substantially long period of time since there are 1214∼6421 mutations in these subclones. while the competing independent minor clones (blue clones in Fig. 2) with 5181∼8194 mutations probably evolved only after the subclones had become highly localized.

*The main clone metastasizing to the liver during early evolution in the primary tumor.* Four metastases in the liver have been sampled and sequenced. The average number and proportion of mutations in the metastases are given in the last two columns of Table 1. It is clear that the metastases originate from Clone 9063 as 98% of its mutations are found in the liver samples. Interestingly, the proportion of the mutations from the pink group in the metastases ranges between 26% and 100% with an average of 72%. In contrast, the four red subclones are rarely present in the metastases with only 1%–5% of the mutations there. This pattern permits us to pinpoint the timing of metastasis to be during the expansion of the pink clones in Fig. 2B, before the red clones began to proliferate. The timing of metastasis corresponds exactly with the cell-movement pattern of Fig. 3.

#### Observations on Case 2: the dominance of a single clone in a large tumor

The main hypothesis of this study is that tumors-in-tumor may not be uncommon. Certainly, it is not expected that every single tumor would have such a complex composition. In fact, at the advanced stage, clonal competition may often reduce the field to a single dominant clone.

**Figure 4.**
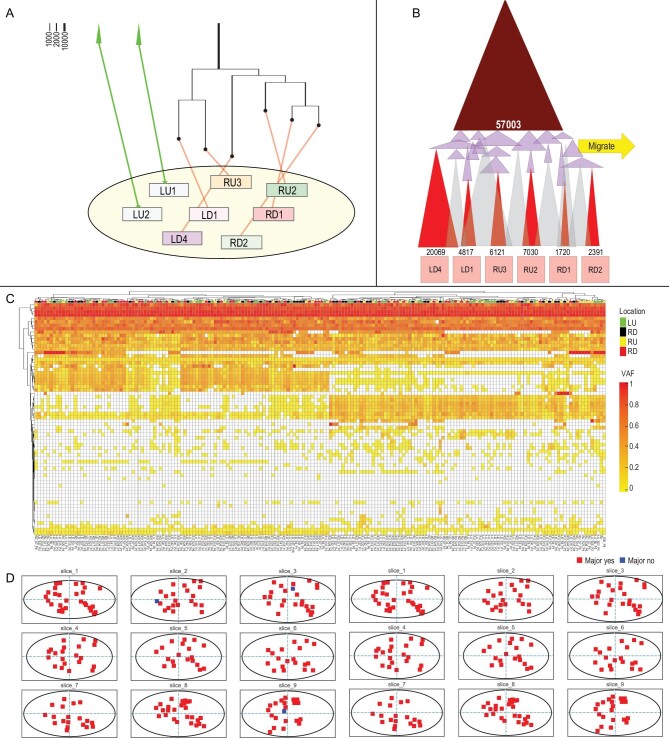
(A and B) The clonal composition of the Case 2 tumor revealed by WGS and target validation. See the descriptions for Fig. 3. (C) Target validation of the Case 2 sample of the primary tumor of four regions (LU: upper left region, RU: upper left region, LD: lower left region, RD: lower right region). (D) The geographical distributions of SNVs across the samples of Case 2.

In the second case of a large colon tumor (∼7 cm in the longest dimension, which has one liver metastatic lesion), we carried out intense search for tumors-in-tumor by obtaining 209 samples from the primary tumor including eight samples used for WGS. Then, 64 variants were validated in all the other samples. Among eight WGS samples of Case 2, all but two samples (LU1 and LU2) are derived from a dominant clone that harbors 57 003 mutations (see Fig. 4 and Supplementary Table S1). This Clone 57003 is present in 98% of the 209 samples. As Fig. 4 shows, Case 2 generally resembles the pattern of Case 1. In particular, the detailed clonal structure of the dominant clone revealed by Fig. 4B is remarkably similar to that of Fig. 2B. The similarities include the many subclones (red) and the intermediate mutational groups (pink), the emergence of these pink mutations marking the time of metastasis to the liver.

Nevertheless, Case 2 differs from Case 1 in two significant aspects. First, the equivalent of the blue clones of Case 1 is entirely missing. These blue clones with high mutation loads and moderate clone sizes are the key evidence of tumors-in-tumor. Second, the ‘green’ minor cell clones are fewer in number and their cells are not dispersed as in Case 1 (details are given in Supplementary Table S1 and Supplementary Fig. S2). In conclusion, the search yields positive results in one of the two cases. The failure to find evidence of tumors-in-tumor in Case 2 does indicate their absence since the sampling is even more intense than in Case 1. The implications of the results in field cancerization [28,35,36] are discussed below.

## DISCUSSION

Tissues may be patchy with some microenvironments being particularly suited to clonal expansion. Factors include mutation rate elevation, inaccessibility to immune cells, blood supplies and a host of other factors [37,38]. Thus, the presence of a tumor could be indicative of a fertile microenvironment for clonal expansion. The view has been generalized into field cancerization [28,35,36], under which a local patch of tissue may have multiple expanding clones. The current study explores the outcome of clonal competition in solid tumors.

The theory presented here explains the not-uncommon observations of multifocal tumors that aggregate but remain anatomically distinct [22–27]. Importantly, the theory also suggests that ‘tumors-in-tumor’ may also be common. While the theory suggests that the phenomenon may be common, the evidence can only be characterized as ‘not so uncommon that it could not be found’. In clonal competition, an aggressive clone bottled in another tumor would quickly approach its size limit as the selective advantage of the clone decreases rapidly when it expands in size. This result is in contrast with the classical theory whereby a mutation with a higher fitness will continue to increase in frequency. In a recent report [3], the more proliferative clones (classified as ‘normal’ in their study) have also been shown to lose to the more abundant but less aggressive clones. The attenuation of selective advantages in clonal competition may be the basis of tumors-in-tumor under field cancerization.

While the genetic diversity within the same tumor has been extensively reported [4,7,39,40], the diversities are generated during the process of clonal expansion from a single progenitor cell. In a single clonal expansion, the closely related subclones are generally neutral in fitness and functionally equivalent [4,6]. In contrast, the independent clonal expansions within the same tumor accrue different sets of mutations and are, hence, far less likely to be functionally equivalent.

Tumors-in-tumor reported here are laborious to identify as very extensive sampling is required for each single tumor. Such sampling is nevertheless highly informative about cell movement during tumor growth. Despite the differences between the two cases studied here, the general patterns of cell movement are very similar. In the rapid growth phase, cells are probably not highly compacted into a small space and are hence relatively free to move about, as well as to metastasize. In this phase, the most aggressive cells are likely to be the winner that takes all. The tumors-in-tumor simulated in Fig. 1 are more likely to emerge in the later phase of tumor growth when cells have much less freedom to move about. The three ‘blue’ clones of Fig. 3 are hence likely to be ‘late-bloomers’ that start to proliferate late in the tumor evolution. In short, the intensive efforts to search for tumors-in-tumor are highly informative about clonal evolution in space and time, even in cases in which tumors-in-tumor are not found.

Finally, tumors inside another larger tumor have no opportunity to show their true tumorigenic potential. Therefore, comparing local relapses with the primary tumor may be a direct test. In particular, we should search for cases with prior evidence of multiple clonal origins in, or next to, the primary tumor but, after the treatment, the relapse is mainly that of the minor clone. Similar evidence has been reported in leukemia although tumors-in-tumor are without meaning in liquid tumor. For solid tumors, an example may be that of the PD8948 case in Yates *et al.* [41] where the genomes of the primary and relapsed tumors show independent origins although additional details are not reported. Hence, the direct test of tumors-in-tumor should be done systematically and will provide valuable guidance to clinical practice including gene-targeting therapy.

## METHODS

### Patient information

The Case 1 patient was a 70-year-old female with a preoperative diagnosis of sigmoid colon moderately differentiated adenocarcinoma with liver metastasis. The patient refused to receive any other treatment except for surgery. She eventually underwent laparoscopic sigmoidectomy. During the operation, four liver lesions were identified and resected, all of which were confirmed to be metastatic liver adenocarcinoma. The postoperative pathology showed that her sigmoid cancer was a 22 × 20 mm protuberant mass and her pathological TNM stage was T2N1cM1. The Case 2 patient was a 35-year-old female. Her preoperative diagnosis was poorly differentiated adenocarcinoma located at the descending colon with liver metastasis. The liver lesion was 21 × 19 mm in size and on the left lobe of the liver. She underwent a radical descending colectomy and the liver lesion was also resected. Her descending colon cancer was shown to be a 70 × 55 mm ulcerative mass. The postoperative pathology confirmed the diagnosis of descending colon adenocarcinoma with liver metastasis, with a TNM stage of T3N0M1. This study was approved by the Institutional Review Board of the Sixth Affiliated Hospital of Sun Yat-sen University. Informed consent was obtained from both of the patients.

### Dense sampling strategy of primary and metastatic tumors

We took paired normal and primary colorectal as well as liver metastatic tumors from two patients and sliced them into a series of contiguous frozen sections that were 0.3 mm thick using the Leica freezing microtome after embedding. Microdissected samples of 0.3 mm in diameter were taken with a micro-punch of inner diameter 0.3 mm. Each cylinder sample contained ∼3000 cells (Supplementary Fig. S1). The coordinates of all samples were recorded for later 3D reconstruction.

In Case 1, 15 (for WGS) + 253(for target sequencing) microdissected samples were taken from the tumors, among which 7 (WGS) + 138 (TS) were from the primary tumor while the others were from liver metastatic lesions. Nine WGS samples and 401 target sequencing samples (including 209 from the primary tumor) were taken from tumors of Case 2. Adjacent normal tissue and blood samples were also taken for control. The *x, y, z* coordinates of all samples were recorded. Genomic DNA was extracted using a Tiangen Micro DNA kit and then subjected to sonication using Covaris. The library was built using the VAHTS TM Universal DNA Library Prep Kit for Illumina.

### Sequencing data processing

After WGS, we filtered out low-quality reads and mapped raw sequencing reads to the GRCh37 human genome with BWA. The filtered reads were then processed through GATK and we then used mutect2 to detect SNVs and filtered out the reads of: (i) prevalent human SNPs, (ii) sequencing depth <10X, (iii) mutated reads number <5 or (iv) >1 SNVs coexisting in 1000-bp length.

### Polymorphic mutations selection and target sequencing

Variants of different groups of mutations (Table 1) discovered in the subset of WGS samples were chosen for verification in the complete set of micro-dissected samples. In Case 1, 106 SNVs were used to genotype 138 primary samples as well as 115 liver metastatic samples. As the tumor in Case 2 was larger than the tumor in Case 1, more samples (209) dissected from the primary and 200 from liver metastasis were used for validation of 64 variants identified by WGS data.

## DATA AVAILABILITY

The data that support the findings of this study are available from the corresponding authors.

## Supplementary Material

nwac250_Supplemental_File
