# Supplementary material for: Very large hidden genetic diversity in one single tumor: evidence for tumors-in-tumor
Source: Natl Sci Rev. 2022 Nov 11;9(12):nwac250. doi: 10.1093/nsr/nwac250 (PMC9869076; doi:10.1093/nsr/nwac250)
Supplement: nwac250_Supplemental_File [file nwac250_supplemental_file.docx]

**Very large hidden genetic diversity in one single tumor**

**– Evidence for tumors-in-tumor**

**SI contents:**

1.Supplemental methods

2.Supplemental Figure S1 and legend

3.Supplemental Table S1 and legend

4.Supplemental references

**Methods**

**WGS data from 7 major samples reveal 13 independent clones in the primary tumor of Case1**

The locations of the 7 samples from the primary tumor (denoted CB, C0, C1, C2, C10, C12 and C14) subjected to WGS are shown in Fig. 2A. The data, summarized in Table 1, are partitioned into groups, highlighted respectively with reddish, blue and green colors. Each row is a set of mutations that exhibit the same geographical pattern, thus forming a clone which is also named after the number of mutations defining the clone.

The reddish group in Table 1 has two sub-groups designated by red (Group 1) and pink (Group 2) colors. These 2 groups of clones are all found in the core samples of CB, C0, C2, C10 (also colored reddish in Fig. 2). Group 3 (colored blue) clones are found in C1, C12 and C14 samples while Group 4 (colored green) are scattered among the seven samples. The distinction of these four groups is informative about the evolutionary dynamics of the tumors.

For Group 1, the 9063 clone is the most dominant clone in size and depth (i.e., number of mutations). Group 1 comprises, in addition to the 9063 clone, 4 other clones that are specific to each of the 4 core samples, CB, C0, C2, C10. These clones (Clone 1214, 3127, 6031 and 6421) are all subclones of Clone 9063 as depicted in Fig. 2 and Table 1. A subclone has all the mutations of the parent clone (in this case, the 9063 mutations) as well as additional ones. These 5 red clones listed on the top of Table 1 are the main clones that dominate the primary tumor. Group 2 clones (colored pink) are also found in the four samples (CB, C0, C2, C10. However, they have far fewer mutations than Group 1 clones and are found in more than one sample. These clones will shed light on the evolution of Group 1 clones, as described in the next section.

Interestingly, 3 of the 7 samples (C1, C12, C14) do not harbor any mutations of the Group 1 or Group 2 clones. Each of the three samples, instead, is dominated by an independent minor clone of Group 3 (Clones 5181, 7506 and 8194; colored in blue). Therefore, the primary tumor depicted in Fig. 2 comprises at least 4 large clones. They are the 9063 clone (plus its 4 subclones in Group 1; all colored red) and the 3 blue-colored clones (Group 3). Furthermore, the clones are scattered widely within the tumor. For example, the clone in C1 is surrounded on all sides by the main clones and are distantly located from other Group 2 clones. The depiction in Fig. 2 thus supports the conjecture of Fig. 1H showing smaller independent clones engulfed by a dominant one.

The last group in Table 1 is the Group 4 clones, colored green. As depicted in Fig. 2, they usually straddle two samples. Group 4 clones appear to be somewhere between clonal expansions in in normal tissues (Lee-Six et al., 2018; Martincorena et al., 2018; Martincorena et al., 2015) and true tumors. As can be seen in Table 1, there are 9 such normal expansions (see also Fig. 2). It is worth noting that these clonal expansions are detected when the cells are found in different samples. For example, Clone 84 are found in C12 and C1 and Clone 66 are present in C12 and C2. In short, the primary tumor is composed of at least 13 clones - one large, 3 medium-sized and at least 9 small clones. Furthermore, we cannot rule out the possibility of many undetected clones that are localized outside of the sample coverage.

**The detailed evolutionary pattern revealed by Group 2 clones (i.e., subclones of Group 1)**

Clones defined by Group 2 mutations (colored pink in Table 1) have the depth of 50 - 700 mutations. They are found in 2 – 3 samples among the 4 major clones (CB, C0, C2, C10). With the sole exception of the 288 clone, they are distributed in a subset of samples of the 9063 clone (see Table 1). A detailed picture is seen more clearly in Fig. 2C across all 138 samples (plus those in the metastases).

To understand Group 2 mutations in relation to the evolution of the primary tumor, we provide a more realistic portrait of the clonal expansion (see Fig. 2). In Fig. 2B, a series subclonal expansions are shown to have occurred between the 9063 clone and its sample-specific sub-clones (i.e., the 6421 group). Group 2 mutations defined this group of intermediate clones (pink color). Therefore, the oldest clone (i.e., 9063) has the widest distribution and the 6421 group of subclones are the youngest as well as the most spatially constrained. Group 2 clones have a spatial distribution in between them since their ages are between the two sets of major clones. For example, the 266 clone as marked can be found in the CB, C0 and C2 samples. In the same framework, each sample comprises more than one clone. Those clones colored in light gray are hypothetical clones, the mutations of which are not identified. Fig. 2B depicts clonal dispersal that resembles “glacier movement”. In this mode, older clones spread more widely and the youngest clones are highly localized.


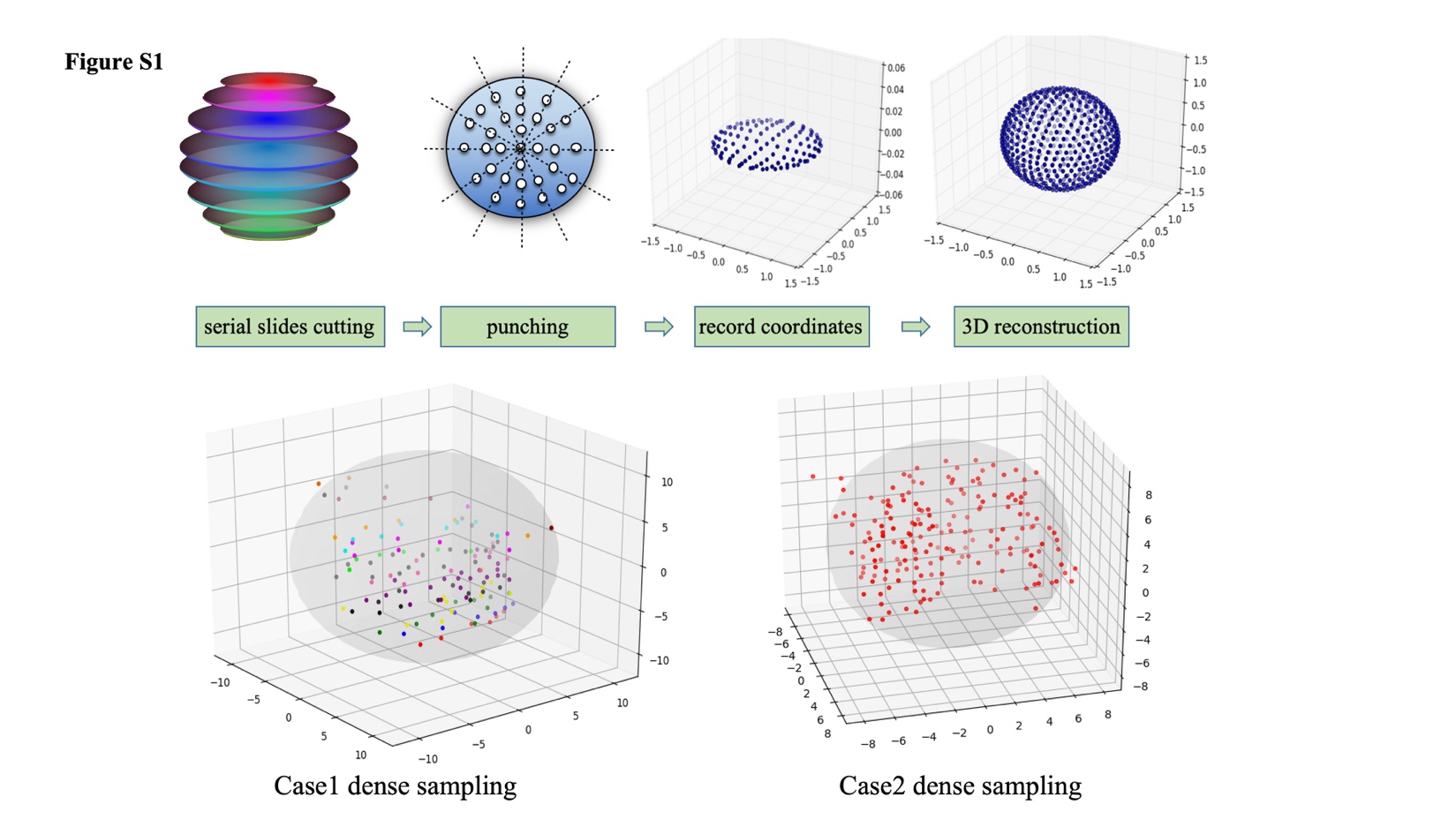


**Figure S1. The sampling scheme in Case 1 and Case 2.**

(A) The tumor is sliced into 0.3mm sections using freezing microtome. Micro-dissected samples, each 0.3mm in diameter, were taken using a micropunch. Each sample cylinder contains ~3000 cells. The sampling sites are evenly distributed. The 3D coordinates of all samples were recorded for later reconstruction. (B) The spatial locations of all samples in Case1 and Case2 tumors are displayed.

**
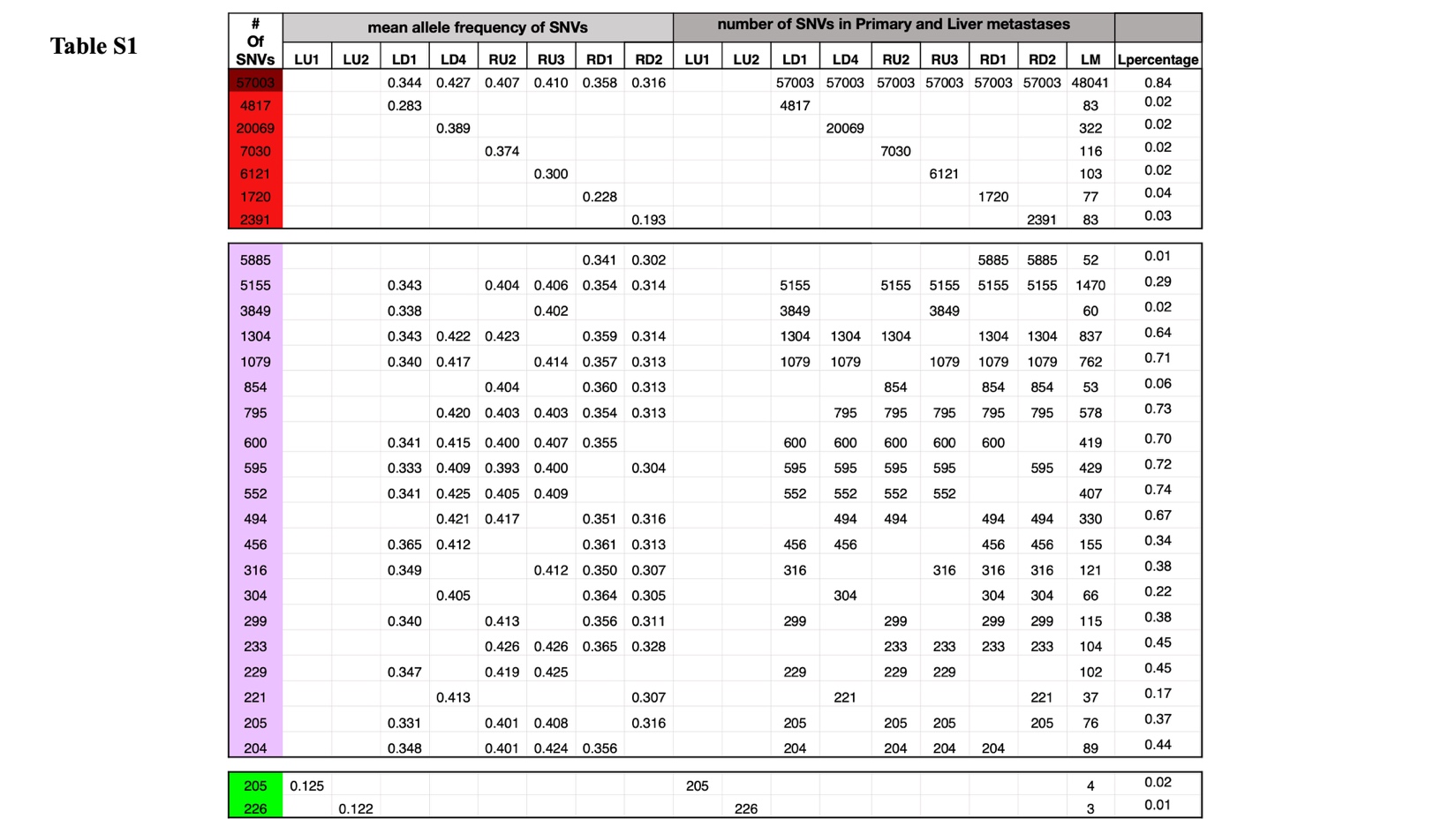
Table S1. Grouping of mutations into clones in Case 2.** See Table 1 section for details.

**Supplemental references**

Lee-Six, H., Ellis, P., Osborne, R.J., Sanders, M.A., Moore, L., Georgakopoulos, N., Torrente, F., Noorani, A., Goddard, M., and Robinson, P.J.B. (2018). The landscape of somatic mutation in normal colorectal epithelial cells. 416800.

Martincorena, I., Fowler, J.C., Wabik, A., Lawson, A.R., Abascal, F., Hall, M.W., Cagan, A., Murai, K., Mahbubani, K., and Stratton, M.R.J.S. (2018). Somatic mutant clones colonize the human esophagus with age. *362*, 911-917.

Martincorena, I., Roshan, A., Gerstung, M., Ellis, P., Van Loo, P., McLaren, S., Wedge, D.C., Fullam, A., Alexandrov, L.B., Tubio, J.M., et al. (2015). Tumor evolution. High burden and pervasive positive selection of somatic mutations in normal human skin. Science *348*, 880-886. 10.1126/science.aaa6806.
